# Prognostic value of cardio-hepatic-skeletal muscle syndrome in patients with heart failure

**DOI:** 10.1038/s41598-020-80641-2

**Published:** 2021-02-12

**Authors:** Takumi Noda, Kentaro Kamiya, Nobuaki Hamazaki, Kohei Nozaki, Takafumi Ichikawa, Takeshi Nakamura, Masashi Yamashita, Shota Uchida, Emi Maekawa, Jennifer L. Reed, Minako Yamaoka-Tojo, Atsuhiko Matsunaga, Junya Ako

**Affiliations:** 1 Department of Rehabilitation Sciences, Kitasato University Graduate School of Medical Sciences, Sagamihara, Japan; 2 Department of Rehabilitation, School of Allied Health Sciences, Kitasato University, Sagamihara, 1-15-1 Kitasato, Minami-ku, Sagamihara, Kanagawa 252-0375 Japan; 3 Department of Rehabilitation, Kitasato University Hospital, Sagamihara, Japan; 4 Department of Cardiovascular Medicine, Kitasato University School of Medicine, Sagamihara, Japan; 5 Exercise Physiology and Cardiovascular Health Lab, Division of Cardiac Prevention and Rehabilitation, University of Ottawa Heart Institute, Ottawa, Canada; 6 Faculty of Medicine, University of Ottawa, Ottawa, Canada; 7 School of Human Kinetics, Faculty of Health Sciences, University of Ottawa, Ottawa, Canada

**Keywords:** Biomarkers, Cardiology, Gastroenterology, Medical research

## Abstract

Although heart failure (HF) and liver dysfunction often coexist because of complex cardiohepatic interactions, the association between liver dysfunction and physical dysfunction, and between coexistence of both and prognosis in HF patients remains unclear. We reviewed 895 patients with HF (mean age, 69.4 ± 14.2 years) who underwent liver function test using model for end-stage liver disease excluding international normalized ratio (MELD-XI) score and physical function test (grip strength, leg strength, gait speed, and 6-min walking distance [6MWD]). In the multiple regression analysis, MELD-XI score was independently associated with lower grip strength, leg strength, gait speed, and 6MWD (all *P* < 0.001). One hundred thirty deaths occurred over a median follow-up period of 1.67 years (interquartile range: 0.62–3.04). For all-cause mortality, patients with high MELD-XI scores and reduced physical functions were found to have a significantly higher mortality risk even after adjusting for several covariates (grip strength, hazard ratio [HR]: 3.80, *P* < 0.001; leg strength, HR: 4.65, *P* < 0.001; gait speed, HR: 2.49, *P* = 0.001, and 6MWD, HR: 5.48, *P* < 0.001). Liver dysfunction was correlated with reduced physical function. Moreover, the coexistence of lower physical function and liver dysfunction considerably affected prognosis in patients with HF.

## Introduction

Heart failure (HF) is a serious public health issue, the incidence of which is increasing around the world^1^. A number of prognostic markers have been shows to predict death and rehospitalization in patients with HF, but their clinical applicability is limited and precise risk stratification remains challenging^1,2^.

Liver dysfunction in patients with HF has been reported to be an important determinant of prognosis, and has recently attracted attention as a cardio-hepatic syndrome^3,4^. The model for end-stage liver disease (MELD) score, which was developed to measure liver dysfunction in patients with end-stage cirrhosis, has been reported to reflect the prognosis of advanced HF^4,5^. In addition, liver dysfunction is known to cause skeletal muscle dysfunction, such as sarcopenia, and physical dysfunction in patients with liver cancer^6–9^. Therefore, severe motor dysfunction may occur in patients with HF complicated with liver dysfunction. In addition, HF patients with liver dysfunction and motor dysfunction are predicted to have poor prognosis.

This study was performed to examine whether the severity of liver dysfunction in patients with HF is associated with decreased physical function, and to investigate the prognostic impact of combined liver and motor dysfunction in patients with HF.

## Results

### Patient characteristics

**Figure 1:**
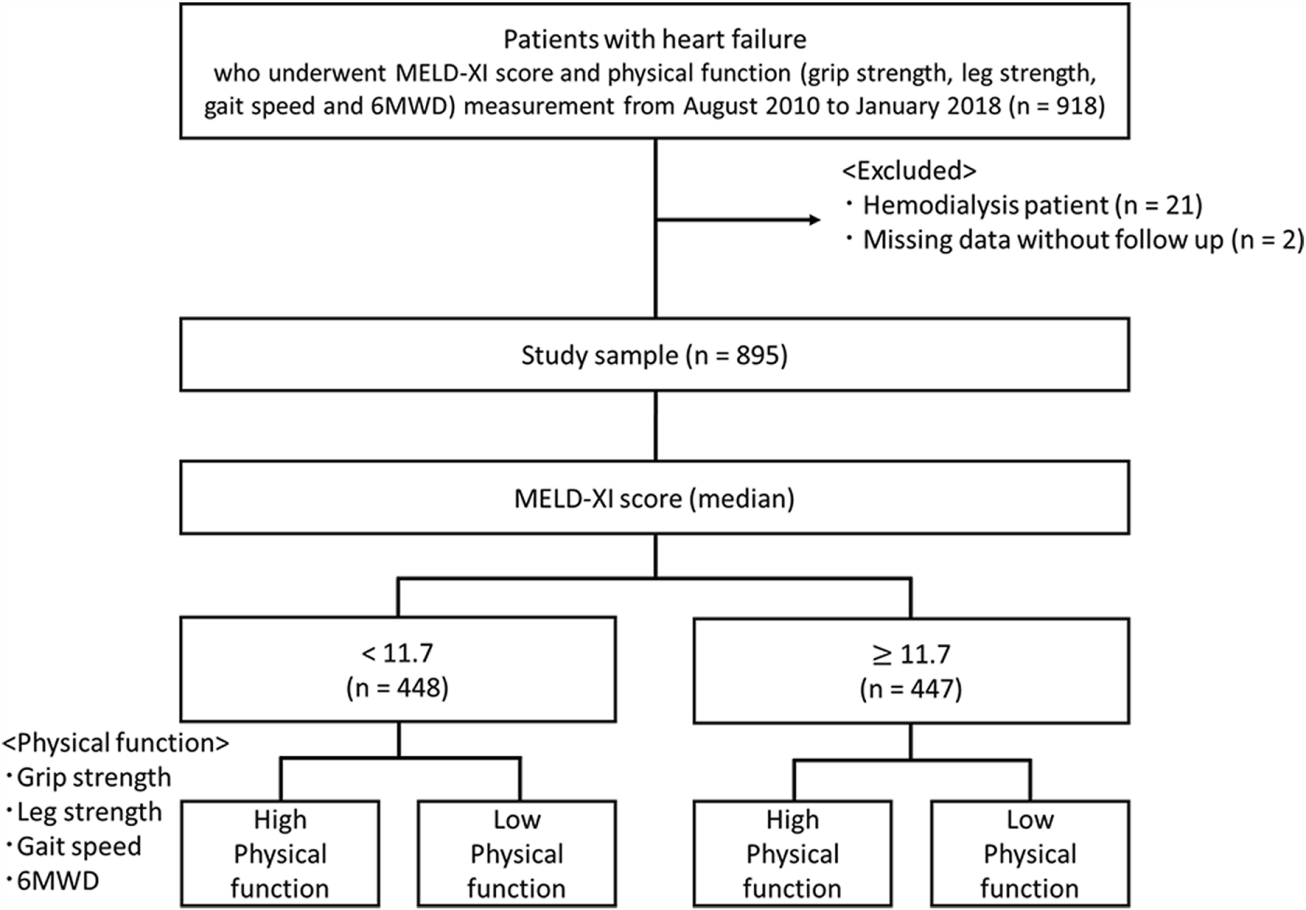
Patient flow diagram. *MELD-XI score* model for end-stage liver disease excluding international normalized ratio score, *6MWD* 6-min walking distance.

A total of 895 HF patients, excluding hemodialysis patients and subjects with missing values, were analyzed of the 918 patients included (Fig. 1). The baseline characteristics of all patients and for groups stratified according to the median of model for end-stage liver disease excluding international normalized ratio (MELD-XI) score are presented in Table 1. The study population had a mean age of 69.4 ± 14.2 years and 60.9% were men. The mean left ventricular ejection fraction (LVEF) was 44.4% ± 17.3% and median B-type natriuretic peptide (BNP) was 665 pg/mL. In comparison to the low MELD-XI score group, participants in the high MELD-XI score group were older, had a higher proportion of males, and had greater HF severity (i.e., higher New York Heart Association [NYHA] classification and BNP) and higher creatinine (Cr), Total bilirubin (T-bil), and prothrombin time-international normalized ratio (PT-INR), and fewer subjects were current smokers.

**Table 1:** Patient characteristics.

	Total	Median of MELD-XI score		
		< 11.7	≥ 11.7	*P*-value
		Low	High	
	*n *= 895	*n *= 448	*n *= 447	
Age (years)	69.4 ± 14.2	67.8 ± 15.4	71.1 ± 12.7	0.001
Male, *n* (%)	545 (60.9)	248 (55.4)	297 (66.4)	0.001
BMI (kg/m^2^)	22.3 ± 4.5	22.2 ± 4.6	22.4 ± 4.4	0.448
Heart rate (beats/min)	82.0 ± 21.7	83.9 ± 22.3	80.1 ± 20.8	0.009
Systolic blood pressure (mmHg)	119.6 ± 30.4	120.4 ± 30.7	118.8 ± 30.2	0.431
Diastolic blood pressure (mmHg)	69.1 ± 20.1	69.8 ± 20.0	68.4 ± 20.3	0.284
NYHA classification, *n* (%), ≥ III	801 (89.5)	391 (87.3)	410 (91.7)	0.038
LVEF (%)	44.4 ± 17.3	45.0 ± 16.7	43.8 ± 17.8	0.318
**Heart failure etiology, *n (%)***				
Ischemic heart disease	303 (33.9)	143 (31.9)	160 (35.8)	0.230
Cardiomyopathy	181 (20.2)	87 (19.4)	94 (21.0)	0.561
Valvular heart disease	144 (16.1)	68 (15.2)	76 (17.0)	0.468
Others	267 (29.8)	150 (33.5)	117 (26.2)	0.019
**Comorbidities**				
Hypertension, *n* (%)	631 (70.5)	307 (68.5)	324 (72.5)	0.213
Dyslipidemia, *n* (%)	398 (44.5)	187 (41.7)	211 (47.2)	0.107
Diabetes mellitus, *n* (%)	581 (64.9)	300 (67.0)	281 (62.9)	0.208
Obesity, *n* (%)	199 (22.2)	91 (20.3)	108 (24.2)	0.173
Atrial fibrillation, *n* (%)	305 (34.1)	131 (29.2)	174 (38.9)	0.002
Current smoker, *n* (%)	163 (18.2)	97 (21.7)	66 (14.8)	0.009
**Medications, n (%)**				
Beta-blocker	692 (77.3)	342 (76.3)	350 (78.3)	0.523
Warfarin	356 (39.8)	156 (34.8)	200 (44.7)	0.003
Aldosterone antagonist	482 (53.9)	237 (52.9)	245 (54.8)	0.592
Statin	397 (44.4)	196 (43.8)	201 (45.0)	0.737
ACE inhibitor or ARB	792 (88.5)	402 (89.7)	390 (87.2)	0.251
**Laboratory examination**				
WBC (×10^3^/μL)	6.2 ± 2.3	6.3 ± 2.3	6.0 ± 2.3	0.022
Hemoglobin (g/dL)	12.5 ± 2.5	13.0 ± 2.3	12.0 ± 2.6	< 0.001
Serum sodium (mEq/L)	138.8 ± 4.8	139.2 ± 4.7	138.5 ± 4.9	0.027
BUN (mg/dL)	27.1 ± 17.6	18.3 ± 6.3	36.0 ± 20.6	< 0.001
Cr (mg/dL)	1.36 ± 0.84	0.88 ± 0.19	1.83 ± 0.96	< 0.001
BNP (pg/mL)	665 (331–1214)	553 (249–1005)	805 (436–1453)	< 0.001
Total bilirubin (mg/dL)	1.2 ± 0.5	1.0 ± 0.1	1.3 ± 0.7	< 0.001
AST (U/L)	31 (22–48)	30 (22–44)	32 (22–50)	0.327
ALT (U/L)	22 (14–39)	23 (15–40)	21 (13–39)	0.252
PT-INR	1.7 ± 1.1	1.5 ± 0.9	1.9 ± 1.3	< 0.001
**Physical function**				
Grip strength (kg)	24.3 ± 9.3	24.8 ± 9.6	23.9 ± 8.9	0.126
Leg strength (%BM)	38.8 ± 14.1	40.6 ± 15.3	37.0 ± 12.7	< 0.001
Gait speed (m/s)	0.98 ± 0.30	1.02 ± 0.30	0.95 ± 0.30	< 0.001
6MWD (m)	355 ± 138	383 ± 133	328 ± 137	< 0.001
All-cause mortality (%)	130 (14.5)	39 (8.7)	91 (20.4)	< 0.001

Mean ± SD; median [interquartile range]; *n*, number (%).

*MELD-XI score* model for end-stage liver disease excluding international normalized ratio score, *BMI* body mass index, *NYHA* New York Heart Association, *LVEF* left ventricular ejection fraction, *ACE* angiotensin converting enzyme, *ARB* angiotensin receptor blocker, *WBC* white blood cells, *BUN* blood urea nitrogen, *Cr* creatinine, *BNP* B-type natriuretic peptide, *AST* aspartate aminotransferase, *ALT* alanine aminotransferase, *PT-INR* prothrombin time-international normalized ratio, *BM* body mass, *6MWD* 6-min walking distance.

### Association of severity of liver damage with physical function

Table 2 outlines the results of multivariate linear regression analyses to evaluate the associations of physical function (grip strength, leg strength, gait speed, and 6-min walking distance [6MWD]) with the severity of liver damage. Each MELD-XI score was associated with reduced predicted physical function measures, after adjusting for covariates (grip strength, β: − 0.106, *P* < 0.001, leg strength, β: − 0.152, *P* < 0.001, gait speed, β: − 0.181, *P* < 0.001 and 6MWD, β: − 0.178, *P* < 0.001). Similarly, BNP levels were associated with leg strength (β: − 0.068, *P* = 0.029), gait speed (β: − 0.084, *P* = 0.007) and 6MWD (β: − 0.114, *P* < 0.001). In addition, hierarchical multivariate linear regression analysis revealed that adding MELD-XI scores or BNP explained additional variance in the physical function measures. The clinical model with MELD-XI score changed the amount of the variance in the physical function measures (grip strength, change in *F*: 27.105, *P* < 0.001; leg strength, change in *F*: 33.980, *P* < 0.001; gait speed, change in *F*: 22.826, *P* < 0.001; and 6MWD, change in *F*: 59.193, *P* < 0.001). The clinical model with BNP changed the amount of variance in the physical function measures (grip strength, change in *F*: 5.811, *P* = 0.016; leg strength, change in *F*: 11.890, *P* < 0.001; gait speed, change in *F*: 13.835, *P* < 0.001; and 6MWD, change in *F*: 22.826, *P* < 0.001). The MELD-XI scores added to the clinical model was significantly more predictive using all physical function measures than BNP added to the clinical model.

**Table 2:** Associations of severity of liver damage with physical function.

Effect	Grip strength		Leg strength		Gait speed		6MWD	
	β	*P*-value	β	*P*-value	β	*P*-value	β	*P*-value
MELD-XI score	− 0.106	< 0.001	− 0.152	< 0.001	− 0.181	< 0.001	− 0.178	< 0.001
Age	− 0.332	< 0.001	− 0.323	< 0.001	− 0.428	< 0.001	− 0.508	< 0.001
Sex (female)	− 0.532	< 0.001	− 0.355	< 0.001	− 0.233	< 0.001	− 0.232	< 0.001
BMI	0.235	< 0.001	− 0.103	0.024	0.003	0.941	0.002	0.955
BNP	− 0.030	0.209	− 0.068	0.029	− 0.084	0.007	− 0.114	< 0.001
NYHA	− 0.052	0.018	− 0.065	0.023	− 0.053	0.065	− 0.018	0.502
LVEF	− 0.078	< 0.001	− 0.047	0.127	0.009	0.756	− 0.035	0.217
Hypertension	0.044	0.059	− 0.029	0.341	0.017	0.579	0.030	0.289
Dyslipidemia	0.028	0.224	0.036	0.238	0.042	0.159	0.017	0.536
Diabetes mellitus	0.002	0.923	0.003	0.909	0.014	0.613	0.018	0.490
Obesity	− 0.066	0.046	− 0.101	0.021	− 0.082	0.058	− 0.105	0.009
Current smoker	0.054	0.019	0.023	0.445	0.053	0.082	0.025	0.378

β, standardized regression coefficient.

*6MWD* 6-min walking distance, *MELD-XI score* model for end-stage liver disease excluding international normalized ratio score, *BMI* body mass index, *BNP* B-type natriuretic peptide, *NYHA* New York Heart Association, *LVEF* left ventricular ejection fraction.

### Prognostic value of MELD-XI score and physical function in patients with HF

**Figure 2:**
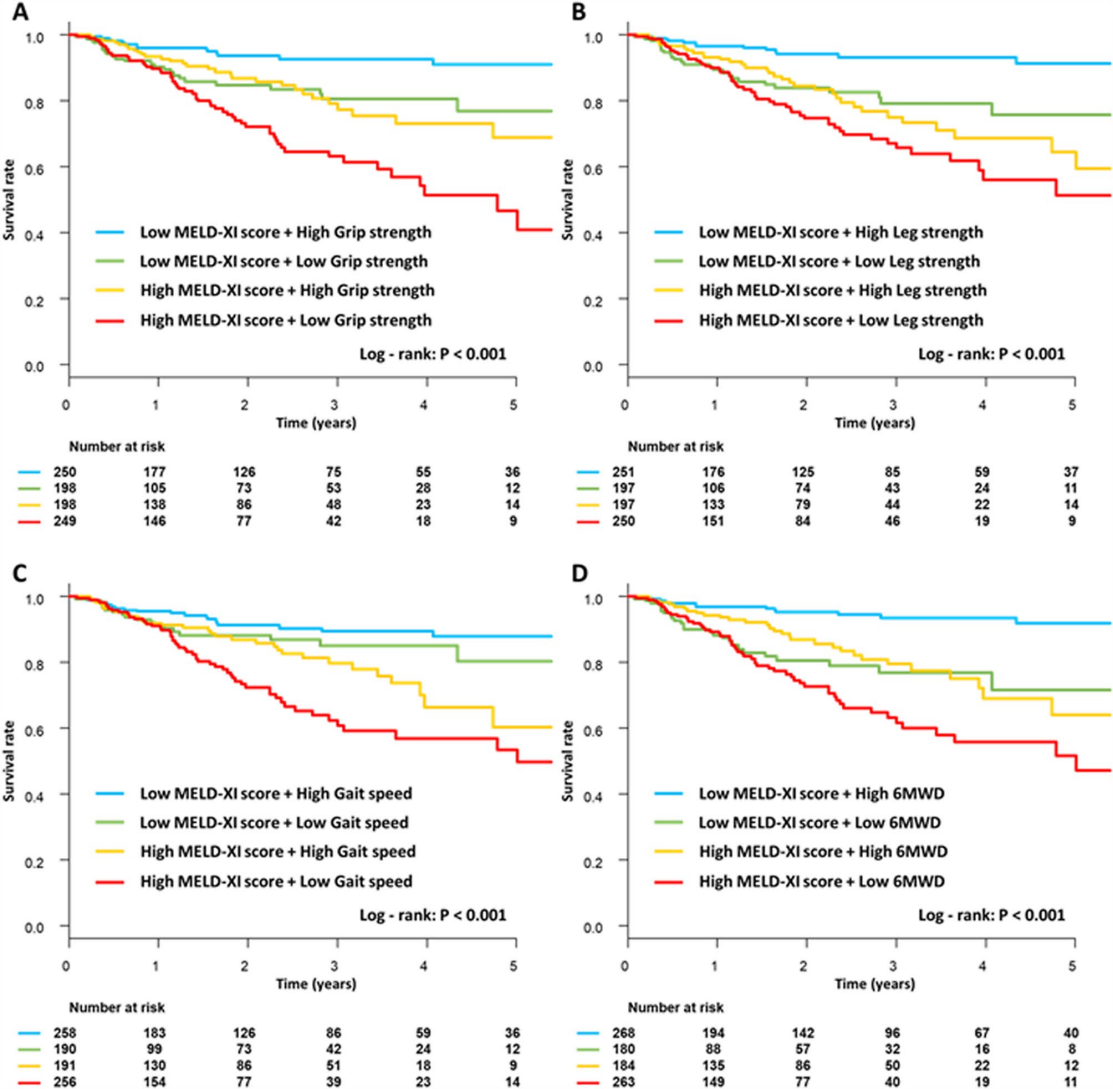
Kaplan–Meier survival curves of overall survival rate in patients divided into four groups based on the median MELD-XI score (11.7) as the cutoff for high and low MELD-XI score and median physical function level for high and low physical function. The physical function parameters used for the analysis were grip strength (**A**), leg strength (**B**), gait speed (**C**), and 6MWD (**D**). *MELD-XI score* model for end-stage liver disease excluding international normalized ratio score, *6MWD* 6-min walking distance.

One hundred thirty deaths occurred over a median follow-up period of 1.67 years (interquartile range [IQR]: 0.62–3.04). Figure 2 shows the Kaplan–Meier survival curves for all-cause mortality in the four groups. The results indicated poorer survival in high MELD-XI score and low physical function group compared to the low MELD-XI score and high physical function group (log-rank, *P* < 0.001 for all). The survival of the low MELD-XI score and low physical function group was similar to that of the high MELD-XI score with low physical function group.

**Figure 3:**
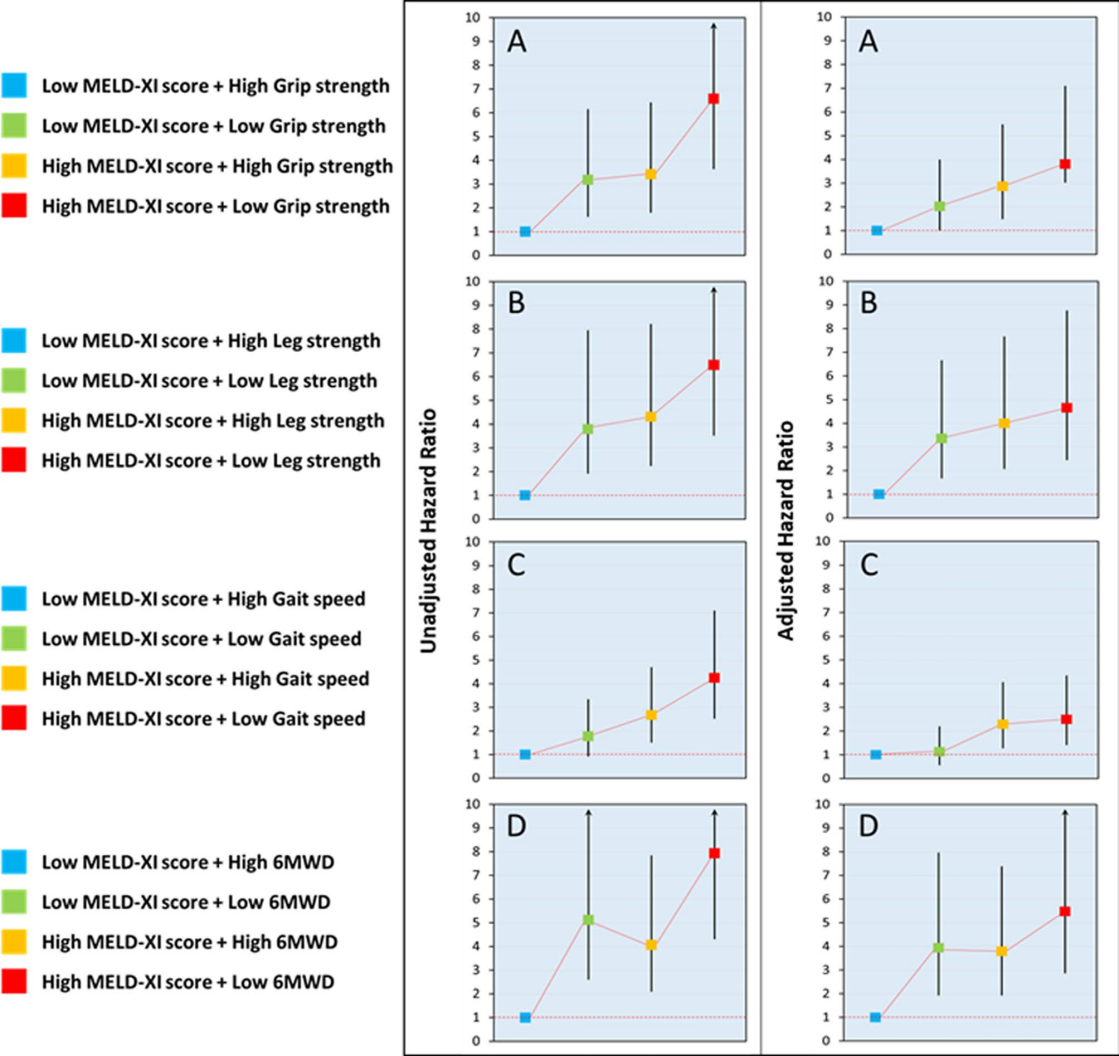
Unadjusted hazard ratio and adjusted hazard ratio* for all-cause mortality in heart failure patients with low or high MELD-XI scores and high or low grip strength (**A**), leg strength (**B**), gait speed (**C**), or 6MWD (**D**). *Adjusted for age, sex, body mass index, left ventricular ejection fraction, New York Heart Association classification, B-type natriuretic peptide, hypertension, dyslipidemia, diabetes mellitus, obesity, and current smoker. Error bars represent 95% confidence intervals. *MELD-XI score* model for end-stage liver disease excluding international normalized ratio score, *6MWD* 6-min walking distance.

Table 3 and Fig. 3 shows the results of univariate and multivariate Cox regression analyses for all-cause mortality. The combination of high MELD-XI score and low physical function measures was a significant predictor of all-cause mortality after adjusting for age, sex, body mass index (BMI), LVEF, NYHA classification, BNP, hypertension, dyslipidemia, diabetes mellitus, obesity, and current smoking. Cox regression analyses revealed that the unadjusted hazard ratios (HRs) between high MELD-XI score and low grip strength (Fig. 3A), high MELD-XI score and low leg strength (Fig. 3B), high MELD-XI score and low gait speed (Fig. 3C), and high MELD-XI score and low 6MWD (Fig. 3D) groups were 6.59 (95% confidence interval [CI] 3.66–11.88, *P* < 0.001), 6.49 (95% CI 3.54–11.92, *P* < 0.001), 4.25 (95% CI 2.54–7.09, *P* < 0.001), and 7.93 (95% CI 4.33–14.52, *P* < 0.001), respectively, compared to the HRs between low MELD-XI score and each high physical function measures. Alternatively, the adjusted HRs between high MELD-XI score and low grip strength, high MELD-XI score and low leg strength, high MELD-XI score and low gait speed, and high MELD-XI score and low 6MWD groups were 3.80 (95% CI 2.04–7.08, *P* < 0.001), 4.65 (95% CI 2.47–8.75, *P* < 0.001), 2.49 (95% CI 1.43–4.33, *P* = 0.001), and 5.48 (95% CI 2.88–10.41, *P* < 0.001), respectively, compared to the HRs between low MELD-XI score and each high physical function measures after adjusting for age, sex, BMI, BNP, NYHA classification, LVEF, hypertension, dyslipidemia, diabetes mellitus, obesity, and current smoking.

**Table 3:** Unadjusted hazard ratio and adjusted hazard ratio for all-cause mortality.

	Unadjusted			Adjusted		
	HR	95% CI	*P*-value	HR	95% CI	*P*-value
Low MELD-XI score + high grip strength	1.00	[Reference]		1.00	[Reference]	
Low MELD-XI score + low grip strength	3.18	1.65–6.13	< 0.001	2.02	1.02–3.98	0.042
High MELD-XI score + high grip strength	3.41	1.82–6.41	< 0.001	2.86	1.50–5.50	0.001
High MELD-XI score + low grip strength	6.59	3.66–11.88	< 0.001	3.80	2.04–7.08	< 0.001
Low MELD-XI score + high leg strength	1.00	[Reference]		1.00	[Reference]	
Low MELD-XI score + low leg strength	3.79	1.94–7.39	< 0.001	3.36	1.69–6.65	< 0.001
High MELD-XI score + high leg strength	4.31	2.27–8.19	< 0.001	4.00	2.09–7.65	< 0.001
High MELD-XI score + low leg strength	6.49	3.54–11.92	< 0.001	4.65	2.47–8.75	< 0.001
Low MELD-XI score + high gait speed	1.00	[Reference]		1.00	[Reference]	
Low MELD-XI score + low gait speed	1.77	0.94–3.33	0.075	1.13	0.59–2.18	0.708
High MELD-XI score + high gait speed	2.68	1.53–4.68	< 0.001	2.29	1.30–4.05	0.004
High MELD-XI score + low gait speed	4.25	2.54–7.09	< 0.001	2.49	1.43–4.33	0.001
Low MELD-XI score + high 6MWD	1.00	[Reference]		1.00	[Reference]	
Low MELD-XI score + low 6MWD	5.12	2.62–10.01	< 0.001	3.93	1.95–7.94	< 0.001
High MELD-XI score + high 6MWD	4.07	2.11–7.82	< 0.001	3.79	1.95–7.37	< 0.001
High MELD-XI score + low 6MWD	7.93	4.33–14.52	< 0.001	5.48	2.88–10.41	< 0.001

Adjusted by age, sex, body mass index, B-Type natriuretic peptide, New York Heart Association classification, left ventricular ejection fraction, hypertension, dyslipidemia, diabetes mellitus, obesity and current smoking.

*HR* hazard ratio, *CI* confidence interval, *MELD-XI score* model for end stage liver disease excluding international normalized ratio score, *6MWD* 6-min walking distance.

## Discussion

There were two major findings in the present study. First, liver dysfunction was shown to be associated with physical dysfunction in patients with HF, even after adjusting for severity of HF, such as NYHA classification, BNP, coronary risk factors, and LVEF. In addition, liver dysfunction was more closely related to physical dysfunction than BNP, an established biomarker of HF. Second, the coexistence of lower physical function and liver dysfunction had a greater impact on prognosis than either alone in patients with HF. These findings suggest the importance of the cardio-hepatic-muscle relation in the prognosis and quality of life of patients with HF.

Previous studies showed that liver dysfunction is the strongest prognostic factor in HF^10^. Moreover, recent reports have shown that the MELD score is correlated with cardiac dysfunction and is associated with prognosis^4,5^. However, little is known about the association between liver dysfunction and physical function in patients with HF. A number of previous studies in patients with cirrhosis and liver disease have reported that sarcopenia or loss of skeletal muscle mass is a common complication and a strong prognostic predictor. The liver plays a major role in carbohydrate, fat, protein, and vitamin metabolism, in addition to lipid transport and bile secretion and excretion, all of which are involved in muscle and protein metabolism and are crucial for nutritional status^7^. Liver dysfunction has been reported to reduce clearance of toxic substances, such as ammonia^11^, and the presence of toxic substances increases skeletal muscle myostatin levels, leading to the progression of skeletal muscle wasting^12,13^. This results in muscle breakdown, and may lead to muscle wasting and reduced physical function^6–8^. These mechanisms may explain the relationships between the combination of liver dysfunction and physical dysfunction and prognosis observed in this study.

In addition, the mechanism of muscle breakdown due to inflammatory cytokines and increased metabolic abnormalities has been reported in patients with HF^14^. In fact, in the present study, BNP and NYHA, which are related to the severity of HF, were also shown to be related to physical dysfunction, similar to the MELD-XI score. These results suggest that the coexistence of liver dysfunction and high severity of HF has a strong impact on reduced physical function.

Our study demonstrated that the complications of liver and physical dysfunction exacerbate the prognosis of patients with HF. Although various possible mechanisms have been postulated to explain these observations, we propose the concept of “cardio-hepatic-skeletal muscle syndrome,” defined as a vicious cycle in patients with HF in which HF causes both hepatic and motor dysfunction^3,15^. Liver dysfunction is a risk factor that increases the severity of HF^10,16^, and it may cause a decrease in metabolism and an increase in utilization of calories from proteins, resulting in skeletal muscle breakdown^6,7^.

Endurance and resistance training have beneficial effects on the cardiovascular system. Myokines (e.g., IL-6 and IL-15), produced and secreted through muscle contraction, regulate the maturation and redistribution of natural killer cells and promote the release of antiinflammatory cytokines, and may therefore protect the cardiovascular system and hepatocytes^17,18^. These myokines are produced exponentially through sustained muscle contraction, such as physical activity^19^, and are involved in determining the lower limb muscle strength and walking speed^20,21^. Irisin, a recently discovered exercise-induced myokine, was shown to inhibit mitochondrial fission and promote biosynthesis, and to be involved in the reduction of oxidative stress thus protecting the heart and hepatocytes during ischemia in mice^22,23^. Furthermore, Akt1 and follistatin-like 1 (FSTL1) are upregulated by skeletal muscle hypertrophy in mice, although these myokines show protective effects on the blood vessels, heart, and cardiovascularization, and are therefore prognostic factors in patients with HF^24,25^. Skeletal muscle loss is correlated with myocardial loss in the presence or absence of heart disease, and may indicate a decrease in muscle cells in other important organs^26^.

Through these mechanisms, liver and motor dysfunction may exacerbate HF, which would thereby further exacerbate liver and motor dysfunction.

Liver dysfunction is associated with poor prognosis in patients with HF^4,16^. The results of the present study showed that severity of liver damage was associated with decreased physical function. MELD-XI scores are determined from biochemical data of T-bil and Cr, which are commonly used in clinical practice and are readily available^4,8^. Therefore, evaluation using the MELD-XI score may provide additional information related to physical function, such as sarcopenia and frailty, in addition to predicting mortality risk in patients with HF. In fact, some studies have suggested that serum bilirubin level is correlated with physical function parameters, such as hand grip strength and 6MWD, and also with quality of life^27,28^. Conventionally, liver function and physical function have been assessed separately in patients with HF. However, a comprehensive assessment may provide additional information to predict future prognosis. Studies in patients with liver disease have shown that the incorporation of sarcopenia into the MELD score provided added value in predicting mortality^29^. Therefore, our findings may extend the treatment and risk management of patients with HF with hepatic dysfunction to interventions addressing cardio-hepatic-skeletal muscle interactions.

This study had some limitations, first, this was a retrospective observational study performed in a single center with a limited follow-up. Second, because this study used only one point of laboratory examination values obtained at the time of hospital visits, the impact of changes in liver function on motor function and prognosis is unknown. In addition, limited information was available regarding drugs prescribed by the attending physician. Therefore, this study did not consider congestion or dehydration as possible confounding factors. However, it has been reported that the components of MELD-XI score (T-bil and Cr) are likely to change depending on the treatment of heart disease, and that the MELD-XI score is likely to change^4^. Therefore, laboratory examination values after acute treatment may include confounding factors due to the initial treatment. Previous studies indicated that acute changes in liver function at the onset of HF are associated with prognosis in patients with HF^30^. This study showed that acute changes at the onset of HF are useful for predicting physical decline and mortality risk. Finally, there was no additional information on echography or diagnostic imaging of the liver. Therefore, organ manifestations due to liver congestion are unclear. However, this was also a strength of our study indicating the utility of MELD-XI score, which can easily evaluate liver function in daily clinical practice.

## Conclusions

The severity of liver damage determined using the MELD-XI score was correlated with reduced physical function. Furthermore, the MELD-XI score was a better predictor of grip strength, leg strength, gait speed, and 6MWD than BNP. Moreover, the coexistence of reduced physical function and liver dysfunction considerably affected prognosis in patients with HF. Taken together, the results of the present study suggested that assessment of liver function and physical function in patients with HF would be useful for guiding treatment and improving patient survival and quality of life.

## Methods

### Patients

The present study used data from the Kitasato University Cardiac Rehabilitation Database. We conducted a retrospective review of 895 consecutive patients with acute HF, defined by the Framingham Criteria^31^, admitted to the Kitasato University Hospital Cardiovascular Center and evaluated for the severity of liver damage, defined according to the MELD-XI score, and physical functions between August 1, 2010 and January 31, 2018. Patients on maintenance dialysis and those with missing data or without sufficient follow-up were excluded from the present study (Fig. 1). The study protocol was performed in accordance with the tenets of the Declaration of Helsinki and was approved by the Ethics Committee of Kitasato University Hospital (KMEO B18-075). Because this study was an observational study that did not involve invasive procedures or interventions, written informed consent was not required under the Japanese Ministry of Health, Labor and Welfare's "Ethical Guidelines for Medical and Health Research for Subjects". Therefore, informed consent was waived with Kitasato University Medical Ethics Organization approval by the institutional guidelines for retrospective observational studies. Participants were informed by opting out that they could refuse to participate because the study protocol was based on the retrospective review of medical records.

### Data collection and assessment of severity of liver damage

The clinical characteristics of the patients, including age, sex, BMI, laboratory examination results, biochemical data, vital signs (blood pressure and heart rate), and echocardiographic data were collected from the electronic medical records at the time of hospitalization. The clinical details of presentation (comorbidities and medication use) were recorded during hospitalization. All-cause mortality was used as the study endpoint, and time to endpoint was calculated as the time from the date of physical function assessment to the occurrence of the event (in days). Follow-up was performed according to discharge and the last day of the period that could be tracked was recorded as the censoring date of the all-cause mortality.

The original MELD score cannot be used in patients taking vitamin K antagonists (oral anticoagulants) because it uses PT-INR values for risk stratification. Therefore, we assessed the severity of liver damage using the MELD-XI score, a model developed by Heuman et al.^32^ that excludes PT-INR, using the equation: MELD-XI = 5.11 × Ln (T-bil) + 11.76 × Ln (Cr) + 9.44. Any variable with a value of < 1.0 was assigned a value of 1.0 to avoid negative scores. Thus, the minimum possible MELD-XI score was 9.44.

### Physical function tests

We measured grip strength, leg strength, gait speed, and 6MWD before discharge from hospital.

A dynamometer was used to measure grip strength (TKK5101 Grip-D; Takei, Tokyo, Japan). Briefly, the patient performed two maximal isometric voluntary contractions of both hands for 3 s each while seated on a bench with the elbow fixed at 90° flexion. The width of the dynamometer handle was adjusted for each patient to match the hand size. The greatest strength measurement in kg was used in the analyses.

The maximal quadriceps isometric strength was measured using a handheld dynamometer to determine leg strength (μ-Tas; ANIMA, Tokyo, Japan). Briefly, with the patient seated in a chair with a non-extensible strap connecting the ankle to a strain gauge, 5-s maximal isometric voluntary contractions of the quadriceps were collected three times for both legs with the hip joint at approximately 90° flexion. Measurements were obtained for the right and left quadriceps consecutively. The greatest strength values on the right and left sides were averaged and expressed as absolute value (kg) and relative to body mass (%BM).

Usual gait speed was measured by timing the patients walking at their usual speed over the middle 10 m of a 16-m walkway. The usual gait speed of each participant was calculated by dividing the distance (in m) by the time (in s).

6MWD was determined according to the guidelines of the American Thoracic Society (ATS) under supervision by technicians^33^. The patients were instructed to walk at their own pace along a straight, flat hallway marked at 1-m intervals, and the distance (in m) was recorded after 6 min.

### Statistical analysis

Normally distributed continuous variables are expressed as the mean ± standard deviation, and non-normally distributed variables are presented as the median (IQR). Categorical variables are expressed as numbers and percentages. Subjects were divided into subcategories according to the median MELD-XI score: < 11.7 points; low group: ≥ 11.7 points; high group. Baseline characteristics were compared using Student’s *t* test, Mann–Whitney U test, or Fisher’s exact test as appropriate.

Independent associations between parameters of physical function (i.e., grip strength, leg strength, gait speed, and 6MWD) were examined by multiple linear regression analysis. The variables entered into the model were defined a priori. Multivariate linear regression analysis was performed to assess the associations of MELD-XI score and the following HF risk factors or severity factors (independent variables) with patient’s predicted physical function (dependent variables): age, sex, BMI, BNP, NYHA classification, LVEF, hypertension, dyslipidemia, diabetes mellitus, obesity, and current smoking. In addition, hierarchical multiple linear regression analyses were performed to assess the contributions of BNP and MELD-XI score to explain continuous levels of physical function after adjusting for clinical model. The clinical model consisted of the variables used in multivariate linear regression excluding BNP and MELD-XI score.

Kaplan–Meier analysis was performed to assess survival, and the log-rank test was used to assess prognostic significance for patients divided into four groups according to the median MELD-XI score (11.7 points) as the cutoff for high and low MELD-XI score and median physical function level for high and low physical function. Parameters of physical function, i.e., grip strength, leg strength, gait speed, and 6MWD, were divided according to the median stratified by sex.

Cox regression analysis was performed to estimate the HR and 95% CI for the prognostic capability of the severity of liver damage, defined by MELD-XI score, and lower physical function by constructing an unadjusted model and adjusted model. An adjusted model of Cox regression analysis was used for the basic attributes of the subjects, severity of HF, and risk factors for HF: age, sex, BMI, BNP, NYHA classification, LVEF, hypertension, dyslipidemia, diabetes mellitus, obesity, and current smoking.

All analyses were conducted using R Studio statistical software (version 3.6.2; R: A language and environment for statistical computing, R Core Team, R Foundation for Statistical Computing, Vienna, Austria, 2019, https://www.R-project.org), with *P* values of < 0.05 used to indicate statistical significance.

## Abbreviations

BMI: Body mass index
BNP: B-type natriuretic peptide
CI: Confidence interval
HF: Heart failure
HR: Hazard ratio
IQR: Interquartile range
LVEF: Left ventricular ejection fraction
MELD-XI: Model for end-stage liver disease excluding international normalized ratio
NYHA: New York Heart Association
PT-INR: Prothrombin time-international normalized ratio
6MWD: 6-Min walking distance
